# Crib-biting

**Published:** 1892-07

**Authors:** 


### CRIB-BITING.

The domestication of the horse, in Western countries at least, has doubtless produced many modifications and great improvements in this most useful animal, physically, and, if I may venture to employ the term, mentally; so that, through long ages of association with man, and skilful breeding and training, as well as feeding and constant use, the creature must now be very different to its wild ancestors. Though its nature and instincts may be essentially the same, yet it has proved sufficiently plastic in the hands of its master to have undergone changes in several respects; and while some special characteristics may have been suppressed or subdued, others have assumed more predominance in the process of evolution from a savage to a highly domesticated animal.

This domestication has not been an unmixed advantage to the horse; for it has entailed changes which, if inevitable, are none the less damaging. These changes are especially related to the animal's robustness of constitution and soundness of texture, and also, to some extent, of habit and instincts. It is liable to many diseases which, there is every reason to believe, have been engendered through influences that attend domestication in our climate; its constitution has been softened and rendered susceptible to the action of agencies which, when it was in an untamed condition, could have had no effect upon it ; while some strange and depreciating habits and vices have become manifested that, there is ample reason for believing, could not have troubled the animal when it was free and as yet unconquered by invincible man.

The bad habits and vices of the horse are somewhat numerous, and the majority of them are well known to horsemen. They are more or less common and frequent, and many of them are due to, or consequent upon, stabulation and stable management. I have mentioned habits and vices, because I am strongly of opinion that such a distinction should be recognized, as it is necessary to mark the difference between what may be a harmless tendency or habit —so far as any deviation from good temper is concerned, and the animal's relations to man and other creatures are involved ; and what may indicate malicious, spiteful, and ungovernable temper— such as kicking, biting, running away, jibbing, rearing, difficult to shoe, harness, or groom, &c.—which is attended with danger to man and beast. Animals which display such serious moral defects as these latter ought to be designated *vicious*, their defects being considered *vices*, in contradistinction to such acquired *habits* as crib-biting, wind-sucking, weaving, trotting in the stable, kicking in the stable when alone at night, lying on the forelegs like a cow, tearing clothing, &c. I am quite aware that some of these are often called vices, and are accordingly estimated as gravely as several of those in the vicious category ; but for several very good reasons a clear distinction between *habits* and *vices* is imperative, as the latter are far more serious than the former, and because they are attended by danger not only to the vicious horses themselves, but to people and animals about them.

Of the *habits* above enumerated, I will select for a few observations that which is, perhaps, the most common, as it certainly is the most curious and troublesome. It may also be said that of all the peculiar habits the horse has acquired in the course of domestication, it is the one that has given rise to most discussion and litigation, while it possesses much interest for physiologists, veterinary surgeons, and horsemen. I am not aware of any other animal than the horse acquiring this habit, which, because of its most characteristic feature, has been named "crib-biting." This, with the closelyallied habit of "wind-sucking," is, so far as I can ascertain, peculiar to solipeds, and perhaps even to the equine species, as I have never heard of an ass being the subject of it. The act itself implies more than the designation would lead one to believe ; for horses will very frequently seize or bite their crib or manger, or anything else accessible, while being groomed or irritated in any way ; but in the habit we are now considering, and which might be looked upon as a habit of self-indulgence, the horse performs the act when alone and undisturbed, and the crib or other body is not seized in a sudden and violent manner, but in a cool, deliberate way, and as if the animal enjoyed it ; and when the seizure has been effected, there takes place a strange physiological phenomenon that even attracts the attention of those who know little of horses. Having fixed the jaws, a sudden, and more or less involuntary and energetic contraction of the muscles of the lower part of the neck takes place—especially of the two muscles that pass from the point of the breastbone to the jaws—as well as some of those of the mouth and body, and coincidently a clucking or belching sound is heard. If the object upon which the horse fixes its mouth is a little low, then the neck is arched and the forelegs are extended. Support on a fixed point or object is essential for the performance of this act ; it need not be the front border of the manger that is so utilized, for horses have been known to crib on the bottom of that receptacle, and on the stall-partition, window-sill, loose-box door, or any other convenient part in the stable ; in harness they have in some cases cribbed on the end of the pole. When nothing else could be got hold of, the head-collar rope or chain has been used when pulled tight. A loose or slack object does not afford support, even a slung or swinging manger is useless to a cribber. It matters not what the object is composed of, so long as it affords a firm support to hold on by or press against while the convulsive but momentary contraction of the muscles takes place, though iron and wood are the materials generally found available. The height of the object is important, that of the manger being the most convenient ; if much higher than this, or even a little lower, the act cannot be performed; on the ground it cannot possibly be done, nor yet when the horse is lying down. There is some variety in the manner in which the act is carried out by different horses. In some cases the object is seized between the full tables of the incisors ; in others it is caught only between the front borders ; in others, again, the mouth is closed, and the front of the incisors—sometimes only the lower, sometimes only the upper ones, but most frequently both—are firmly pressed against the resisting body. These are real cases of crib-biting, and the appearance of the teeth, when the habit has been in operation for any length of time, affords good and reliable evidence of its existence, as the result is characteristic. They are evenly and smoothly worn away, in the great majority of instances, in front, the sharp anterior margin having disappeared by wear, leaving a well-defined bevelled surface. The central incisors are first affected in this way, then those next to them, but it only very rarely happens, and then when the middle teeth are worn nearly to the gums, that the corner incisors are worn in this manner. This condition of the teeth differs from that produced by the horse biting its manger while being groomed or irritated, as then they are chipped on the anterior border, and are consequently rough and irregular on the edge; whereas in cribbers they are worn smooth. In some very extraordinary instances the teeth are not employed at all, and pressure is made by the lips, the chin, or the back of the lower jaw. But in all cases the contraction of the muscles alluded to takes place, and is generally accompanied by the curious gulping or belching sound.

This convulsive act may be repeated at wide intervals or very frequently. At first it is rare, but in the course of time it occurs oftener, and in bad cases only a brief time intervenes between each performance. Some horses have been observed to crib-bite as often as fifteen to eighteen times in a minute, and one authority has counted as many as 295 such acts in an hour. The habit is only indulged in by some horses when they are alone, and no person is in the stables; but these are animals which have been chastised for it, and watch for an opportunity to perform it unobserved. They are usually horses which have been in dealers' stables for some time. Other horses crib only when feeding out of the manger, and others do it when not feeding. Some have been seen to do it when eating straw, and not when feeding on hay or oats; others, again, crib at nearly every mouthful of food, while some only begin when they have finished eating, and others before commencing. Some cease cribbing when strangers are present in the stable, and others begin when some one accidentally appears.

The habit is not always easy to detect at the commencement, especially when the teeth are not worn, or when the horse has been punished for practising it; and some animals have to be quietly watched in the stable for some time before conclusive proof can be obtained. In some the habit is so confirmed and the desire to indulge in it so strong, that when everything upon which they can press their teeth and jaws in the stable is removed, they will endeavor to make use of their fore limbs; and it has been remarked that such persistent crib-biters will even attempt to perform the act while at work—indeed, instances have been recorded of horses that only resorted to it when employed out of doors either when in movement or resting for a brief period, and using the bit as their fixed point. In many instances the horse moves its lips over the object before seizing it, or licks and moistens it with saliva. When the habit is not very confirmed it will often cease—for a time at least—with some change in the animal's surroundings. Removal to another dwelling, lowering or removing the manger, or, if tied up in a stall, moving into a loose-box, will frequently cause the habit to cease for a time. In every regiment of cavalry there are a number of crib-biters, and I have always observed that on active service or manœuvres these have refrained from the habit; in all probability because on picket lines they could not find the needful convenient support for their jaws. A French authority, Bellanger, describes the case of a troop horse —a notorious cribber—which, during the Italian war of 1858, ceased to do so, but began again on returning to its stable in Paris. I have also noted that when crib-biters were ill they refrained from cribbing, and the resumption of the habit was an infallible sign of restoration to health.

An injury to the mouth will often hinder a horse from crib-biting; and horse-copers, when desirous of getting rid of the "cribber" to some unwary purchaser, often have recourse to this stratagem. The most frequent dodge is to drive small wooden or iron pegs between the incisor teeth, and in such a way as not to be easily discovered.

But the intermissions that are marked are usually of short duration, for the habit evidently affords so much satisfaction that it soon becomes a second nature, and if interrupted from some cause is eventually recommenced and continued during life. It is sometimes complicated with the habit of "wind-sucking," which might be considered as crib-biting without support. In this act, which is much more rare than that of crib-biting, the horse usually raises the nose high in the air, moves the lips rapidly up and down, as if sucking, lifts its tongue towards the roof of the mouth, contracts the cheeks, extends the head rigidly and horizontally, and swallows a mouthful of air; but there is seldom much noise accompanying the effort. In other cases of wind-sucking, the lips, cheeks, and tongue are moved in the same manner, but the horse suddenly lowers the head—at times as low as the knees—makes an effort to suck, appears to swallow air, and may or may not make a sound like that heard in crib-biting: But it often happens that the attempt to swallow a mouthful of air is not successful at first, and only some saliva goes down instead ; so that the act continues to be repeated until fully accomplished, and the desire is satisfied.

In some very exceptional instances, horses will crib-bite and suck wind ; though, as we shall see presently, they may also suck wind while crib-biting only, and they may even perform these acts alternately.

The habit of crib-biting is usually observed in horses at adult age, though it has been witnessed at all periods of life. It has been known to commence in foals soon after they were weaned, but it is rarely acquired after fifteen years of age.

I have said that it is a habit peculiar to stabled horses ; but in this I am not quite correct, perhaps, as high authorities assert that it is known nearly everywhere—among the horses of the Tartar nomads as well as those of Arabia ; but I have never noticed it in Mongolian, Manchurian, Japanese, Indian, Syrian, or Arabian horses. It is certainly much more prevalent among high-bred, nervous horses than among under-bred, coarse, lymphatic animals.

How it commences has been a fruitful subject of contention ; some persons maintain that it is a gradually acquired habit, and is due to indigestion ; others that it may be suddenly adopted, and that it is in no way connected with disease, though, like many other habits in man and beast, it may eventually induce a morbid condition. With the opinions of the latter I am inclined to coincide. I have known of a horse acquiring the habit within a week, and have been told of others becoming crib-biters in one or two days. Of course, the habit must have a commencement, and the first seizure of the crib, with the spasmodic contraction of the muscles, is that commencement. This need not take long to develop, and it is well known that excellent horses in the full bloom of health are crib-biters, and may continue so for a long time without the slightest evidence of disease being induced thereby. In itself it is not a disease, nor is it the result of disease ; it cannot, therefore, be considered an unsoundness, though it may in the course of time occasion disease—a very different affair.

It has been known to horsemen from the earliest antiquity, and old writers have viewed it as an imitative habit ; recent observers do the same, though it is generally difficult to discover the origin of a habit of this kind. I have known it to arise from imitation, young horses having picked it up from being placed where they could see a cribber at work ; and experienced veterinarians will bear me out in stating that it sometimes spreads in collections of horses like a contagious disease from this cause alone, and especially if the horses are well bred and fed, and kept in stalls without doing much work. It is interesting to watch a young horse when first placed alongside a crib-biter ; how it regards the latter with astonishment and curious attention, and soon begins to lick the manger ; it is not long before it moves about its jaws, gets hold of the manger now and then with its teeth, and gradually acquires the desired position for its tongue, and often finally succeeds in accomplishing the act—though in a more or less imperfect manner, and in its own way. Hence there are so many modes of crib-biting. I have always endeavored to keep crib-biters by themselves, or, at least, apart from young horses.

It has sometimes been observed that horses which lick the walls of their stables and their mangers, or play with their head-collar rope or chain, become at last crib-biters, and this may be so ; but, as said before, the origin of a habit of this kind is not easily traced in every instance.

Heredity, of course, has been brought into the discussion, but, as in so many other instances, without a sufficient proof. Careful investigation into the history of crib-biters has shown, in fact, that there is no foundation for the statement, which accumulated evidence negatives.

In Prussia I believe the notion still prevails that the habit is hereditary, and a crib-biting stallion is not allowed in the stud. But it was found in the *haras*, at Strasburg, in 1873 and 1874, that a fourth of the number of stallions there had acquired this habit, and yet their progeny was no more affected than that of the sound sires ; while at Benfield Station in the same region there were none other than crib-biting stallions for four years, and their stock remained free from the habit.

It is probable that if horses were kept in box-stalls, and allowed to move about in them, instead of being fixed in stalls with their heads perpetually in one direction, staring at the wall, with their mouths hanging over the manger, there would be far fewer crib-biters, and certain diseases would be less common.

Into the question as to whether horses swallow air or expel it from the stomach in the act of crib-biting, I cannot enter here ; so content myself with stating that there is experimental evidence to show that both occurrences may take place. In some horses the air can be seen descending the neck into the chest like a bolus; these are the horses that often become enormously distended with air, and suffer from flatulent colic. In others the gases from the stomach can be seen ascending in the same way, and the odor of the food or medicine the animals may have had can be perceived in the eructations.

Though horses may indulge in the habit for years without appearing to suffer any inconvenience, yet it is an unpleasant one to look at, and generally, in a longer or shorter period, leads to indigestion, alterations in the structure or the stomach and intestines, loss of condition, tendency to colic, etc.

The most objectionable cribbers are those which indulge in the babit while feeding; as not only do they waste their food, but usually a quantity of saliva escapes from the mouth, and this loss is damaging to digestion. Feeding from a nose-bag will, of course, prevent cribbing at this time.

The many devices to prevent horses crib-biting are so familiar that they need not be referred to. The habit can be prevented, though it can rarely be eradicated when once acquired. The chief object should therefore be to prevent its acquirement.—*Baily's Magazine.*

